# The Monteggia fracture: series of 20 cases

**DOI:** 10.11604/pamj.2014.19.51.5300

**Published:** 2014-09-22

**Authors:** Aniss Chagou, Abdelkarim Rhanim, Mohammed Saleh Berrada

**Affiliations:** 1 Department of Orthopaedic Surgery and Traumatology, University Hospital Center, Ibn Sina, Mohamed V University, Rabat, Morocco

**Keywords:** Monteggia fracture, surgical management, ulna

## Abstract

The Monteggia fracture is one of the pitfalls of conventional diagnosis of upper limb trauma. Through a retrospective study of 20 cases diagnosed at the Department of Orthopaedic Surgery and Traumatology, University Hospital Center, Ibn Sina, Mohamed V University, Rabat, Morocco, between 2010 and 2014, we have tried to do an update on the management of Monteggia fractures either at of paraclinical exams or the surgical management. We support the idea that the dislocation of the radial head should be sought systematically to any isolated fracture of the ulna, for not to miss fracture Monteggia authentic. Rehabilitation of the upper limb must be done as soon as possible.

## Introduction

The Monteggia fracture is defined by the combination of a fracture of the ulna and dislocation of the radial head. Indeed, this lesion was described by Monteggia in 1815 [1], is a classic trap of upper limb trauma, hence the importance of systematically seeking a dislocation of the radial head after the discovery of an isolated fracture ulna. Our study is conducted for an inventory in Avicenne hospital regarding this fracture. This work was conducted to study the Monteggia fracture, both the paraclinical, clinical, and therapeutic, at Department of Orthopaedic Surgery and Traumatology, University Hospital Center, Ibn Sina, Mohamed V University, Rabat, Morocco.

## Methods

Compiled records of 20 cases of Monteggia fractures at Department of Orthopaedic Surgery and Traumatology, University Hospital Center, Ibn Sina, Mohamed V University, Rabat, Morocco, between January 2010 and December 2014. The results were treated using the Microsoft Excel 2003 tool.

## Results

**Figure 1:**
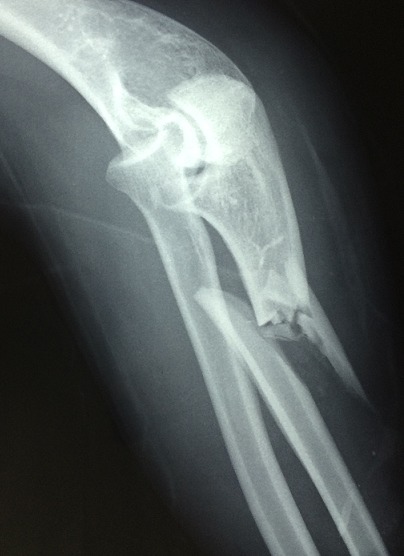
Radiography of monteggia fracture

The average age of patients was 25 years, ranging from 19 years to 42 years. The sex ratio is 16/4 for men. Distribution depending on which side achieved: Both sides, right and left, are achieved in the same proportion. For the circumstances of occurrence: Accidents of the highway in 50% of cases, agression in 40% of cases, and domestic accident in 10% of cases. The Mechanism of injury is direct reception on the forearm in 100% of cases. Clinically the Patients report pain in the forearm and functional impairment of the upper limb in 100% of cases. On examination there is a skin incision in 80% of cases, a deformation of the forearm in 100% of cases. We found no instances of sensorimotor deficit. The Monteggia fracture is isolated in 50% of cases. The radiographs of the elbow and forearm face and profile showed an anterior radial dislocation (Bado type 1) in 80% of cases, and anterolateral (Bado type 3) in 20% of cases (Figure 1).

**Figure 2:**
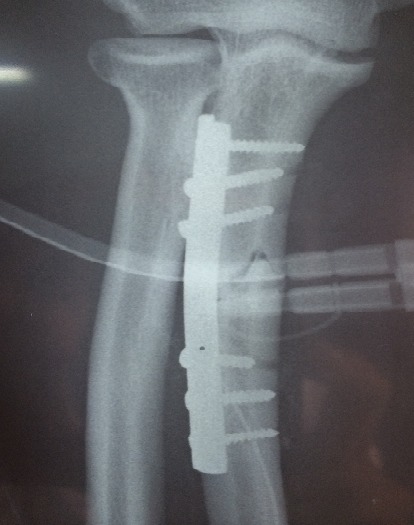
Radiography post operative control

**Figure 3:**
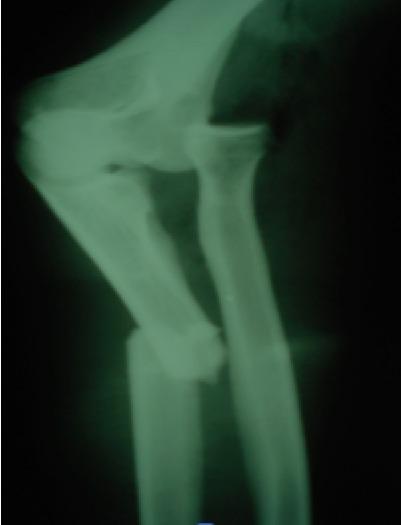
Radiography showing the Monteggia fracture in a second patient

**Figure 4:**
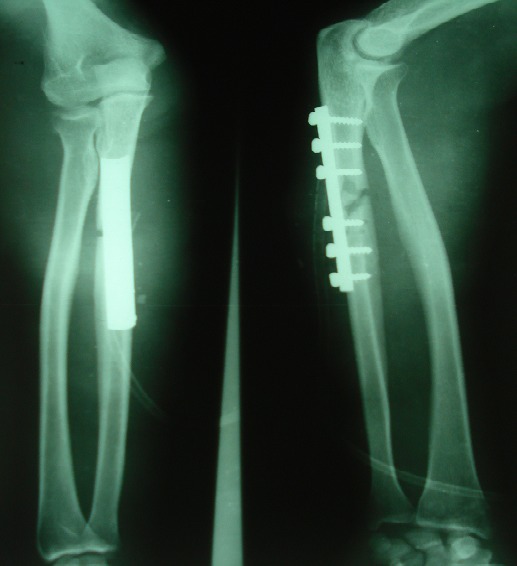
Radiography post operative control of the second patient

All patients were operated under local anesthesia (axillary block), by posterior approach in all cases. Debridement was performed for all open fractures less than 4 hours after the trauma. The treatment of ulnar fracture was provided by plate screws (Figure 2, Figure 3, Figure 4). The reduction of the radial dislocation was made in 19 patients spontaneously. One patient had a dislocated still not reduced after osteosynthesis of the ulna radial head. The patient was reoperated; reduction and osteosynthesis of the ulna were taken. Perfect reduction of the fracture of the ulna was required to reduce the radial head. For all patients, brachiocephalic antébrachiopalmar plaster was manufactured and kept for 6 weeks with the release of the elbow after 3 weeks. Rehabilitation of the elbow was started after the release of the elbow.

## Discussion

Monteggia fracture in our series is a fracture in young adults, with male predominance, which joined the study by Singer which shows a significantly higher fracture of the forearm rate (including fracture Monteggia) in men between 15 to 44 years [2, 3]. The risk of these fractures is five times higher than for a woman over 60 years [4]. This fracture often occurs after a highway accident or aggression, which explains the male predominance of age. The mechanism is straightforward in all patients in our series. In fact, although this mechanism is difficult to identify in highway accidents, it is easier to find in the context of attacks by the classic blow on a forearm placed in position face protection [5, 6]. The clinic did not note specific complications to this fracture, although some studies note an increased rate of neurological complications is an average of 7%, often occurring on the radial nerve, spontaneous recovery occurs most frequently [7]. The skin incision is secondary rule to a high-energy trauma. The diagnosis is essentially by radiographs of the elbow and forearm, face and profile, which show a clear predominance of type 1 Bado, also relating to other international studies [6]. With regard to the treatment, it is ensured by the screw and plate fixation and the reduction of radial dislocation after a posterior approach, with a local anesthesia, in all cases. Indeed, Monteggia fracture requires anatomic reduction of the fracture of the ulna; the bone will promote humeroulnar joint stability by anatomically restoring the congruence between the cup and the radial head of the humerus capitelum [6].

## Conclusion

Dislocation of the radial head should be sought systematically to any isolated fracture of the ulna, for not to miss an authentic fracture Monteggia. Also note that the rehabilitation of the upper limb must be done as soon as possible, when the stability of the elbow permits, to prevent the dreaded elbow stiffness after this type of fracture.
